# Pituitary infiltration by non-Hodgkin's lymphoma: a case report

**DOI:** 10.1186/1752-1947-3-9293

**Published:** 2009-11-24

**Authors:** Gonca Tamer, Ilkay Kartal, Ferihan Aral

**Affiliations:** 1 Division of Endocrinology and Metabolism, Department of Internal Medicine, Istanbul Faculty of Medicine, University of Istanbul, Capa, Istanbul, Turkey

## Abstract

**Introduction:**

Pituitary adenomas represent the most frequently observed type of sellar masses; however, the presence of a rapidly growing sellar tumor, diabetes insipidus, ophthalmoplegia and headaches in an older patient strongly suggests metastasis to the pituitary. Since the anterior pituitary has a great reserve capacity, metastasis to the pituitary and pituitary involvement in lymphoma are usually asymptomatic. Whereas diabetes insipidus is the most frequent symptom, patients can present with headaches, ophthalmoplegia and bilateral hemianopsia.

**Case presentation:**

A 70-year-old woman with no previous history of malignancy presented with headaches, right oculomotor nerve palsy and diabetes insipidus. As magnetic resonance imaging revealed a sellar mass involving the pituitary gland and infundibular stalk, which also extended into the right cavernous sinus and sphenoid sinus, the patient underwent an immediate transsphenoidal decompression surgery. Her prolactin was 102.4 ng/ml, whereas her gonadotropic hormone levels were low. A low level of urine osmolality after overnight water deprivation, along with normal plasma osmolality suggested diabetes insipidus. Histological examination revealed that the mass had been the infiltration of a high grade B-cell non-Hodgkin's lymphoma involving respiratory system epithelial cells. Paranasal sinus computed tomography scanning and magnetic resonance imaging of the thorax and abdomen were performed. Since magnetic resonance imaging did not reveal any abnormality, after paranasal sinus computed tomography was performed, we concluded that the primary lymphoma originated from the sphenoid sinus and infiltrated the pituitary. Chemotherapy and radiotherapy to the sellar area were planned, but the patient died and her family did not permit an autopsy.

**Conclusion:**

Lymphoma infiltration to the pituitary is difficult to differentiate from pituitary adenoma, meningioma and other sellar lesions. To plan the treatment of lymphoma infiltration of the pituitary gland, it must be differentiated from other sellar lesions.

## Introduction

The most frequent tumor of the pituitary gland is pituitary adenoma, but craniopharyngioma, Rathke cleft cyst, dermoid, epidermoid germinoma, metastasis, meningioma, arachnoid cyst, sarcoidosis, tuberculosis, histiocytosis, lymphocytic hypophysitis, schwannoma, infundibular glioma, cavernous carotid artery aneurysm and pituitary involvement in lymphoma or leukemia are included in the differential diagnosis of sellar and parasellar diseases [1,2].

Primary sellar lymphoma is a very rare disease accompanied by acquired immunodeficiency syndromes. The mean age of immunocompetent patients with sellar lymphoma is 60 to 70 years old. Sellar lymphoma is an exceedingly rare disease and is asymptomatic in most cases. Since the pituitary gland has a great reserve capacity, pituitary involvement in lymphoma also appears to be asymptomatic in most cases. In many cases, diabetes insipidus (DI) seems to be the most common clinical presentation as the posterior lobe of the pituitary is supplied with blood directly from the systemic circulation while the anterior lobe is not. In patients with lymphoma, it is essential to differentiate pituitary involvement in lymphoma from benign lesions for the appropriate therapeutic plan. Local therapy aiming at symptom relief may be beneficial [1-3].

## Case presentation

**Figure 1:**
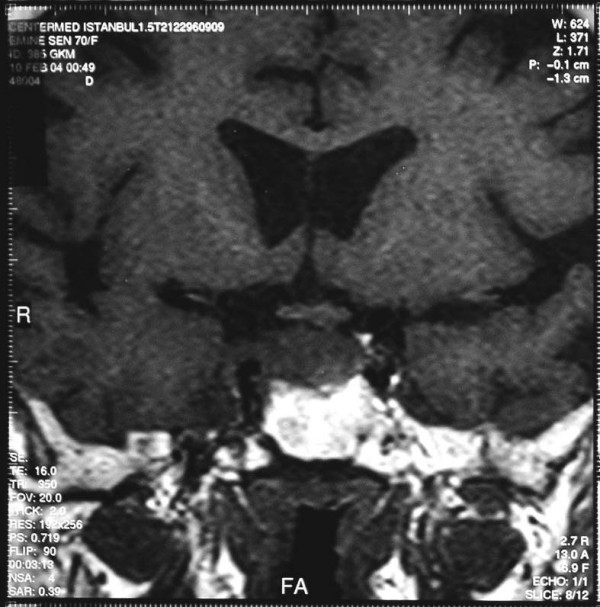
**Precontrast coronal pituitary magnetic resonance imaging T1W1**.

**Figure 2:**
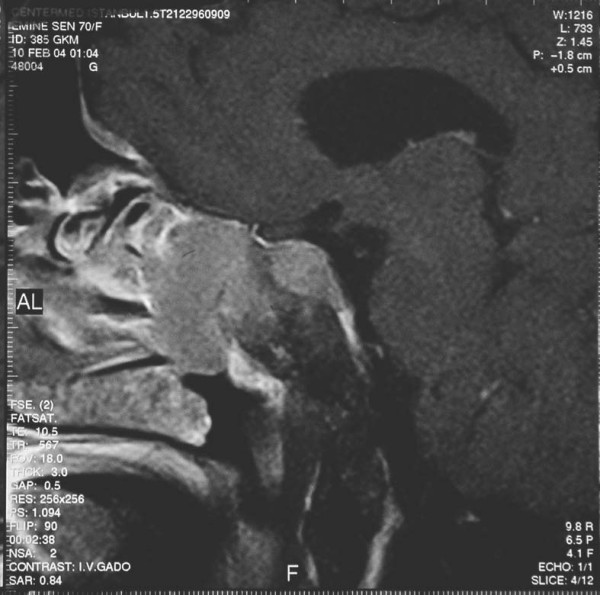
**Sagittal pituitary magnetic resonance imaging T1W1 with contrast agent applied**.

**Figure 3:**
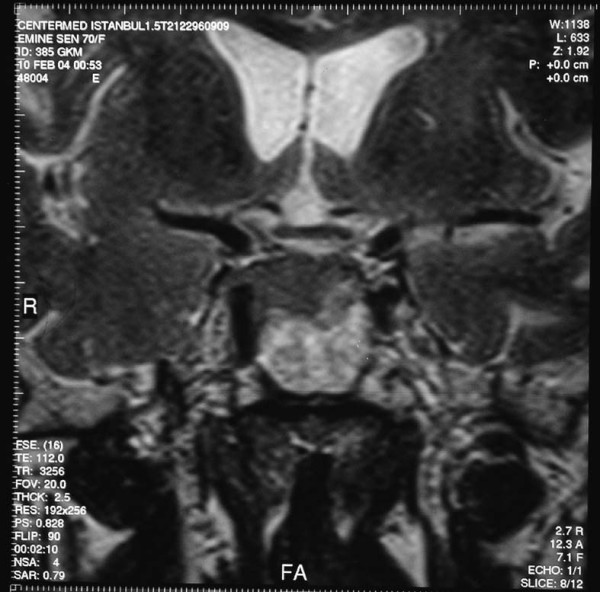
**Coronal pituitary magnetic resonance imaging T2W1 with contrast agent applied**.

A 70-year-old woman presented with headache, weakness, fatigue, right palpebral ptosis and diplopia. Magnetic resonance imaging (MRI) of the sella revealed a sellar mass involving the pituitary gland and infundibular stalk, which also extended into the right cavernous sinus and the sphenoid sinus. The neurologists demonstrated infiltrations of the right II, III, IV, V and VI cranial nerves (figure 1, figure 2 and figure 3).

A week after occurrence of right palpebral ptosis, the patient received immediate transsphenoidal decompression surgery. The histological examination revealed that the mass was the infiltration of a high-grade B-cell non-Hodgkin's lymphoma involving respiratory system epithelial cells.

After the transsphenoidal operation, the patient was transported to our endocrinology clinic. Her skin was pale and she had right palpebral ptosis. No lymphadenopathy or goiter was found. She did not have galactorrhea. Her axillary and pubic hair was scant. Her blood pressure was 120/70 mmHg with a pulse rate of 80 beats/per minute (hypertension and bradycardia existing before the operation, were improved postoperatively). Examination of the chest, abdomen and cardiovascular system was normal.

As per whole blood count, hemoglobin was 12.3 g/dl, hematocrit was 38%, white blood cell count (WBC) was 11,760/mm^3^, lymphocytes were 1400/mm^3^, neutrophils were 9660/mm^3 ^and platelet count was 123,000/mm^3^. The biochemical analysis results of the blood were as follows: glucose: 90 mg/dl, blood urea nitrogen (BUN): 22 mg/dl, creatinine: 0.7 mg/dl, sodium: 141 mEq/L, potassium: 4 mEq/L, serum osmolarity: 295 mOsm/kg H_2_O, with a paired urine osmolality of 173 mOsm/kg, free T4: 20.4 pmol/L, thyroid stimulating hormone (TSH): 0.705 mIU/L, cortisol at 8 am, 16.7 mcg/dl, adrenocorticotrophic hormone (ACTH) at 8 am, 41 ng/L, luteinizing hormone (LH): 0.1 mIU/ml, follicle-stimulating hormone (FSH): 1.8 mIU/ml, estradiol: 8.1 pg/ml, prolactin (PRL): 102.4 ng/dl. Low levels of urine osmolality after overnight water deprivation along with normal plasma osmolality suggested DI. Desmopressin treatment was not administered because the daily urine volume did not exceed 4 liters (her weight was 56 kg).

Hyperprolactinemia was attributed to the stalk compression which interrupted dopamine transport to lactotrophs. The low levels of FSH and LH observed in this post-menopausal woman suggested infiltration of the anterior pituitary by lymphoma. Because primary sellar lymphoma is very rare and histological examination of our patient revealed that the mass was due to infiltration of a high-grade B-cell non-Hodgkin's lymphoma involving respiratory system epithelial cells, computed tomography (CT) of the paranasal sinus and MRI of the thorax and abdomen were performed.

Although MRI of the thorax and abdomen did not show any abnormalities, when the paranasal sinus CT scan was performed, we concluded that the primary lymphoma originated from the sphenoid sinus and infiltrated the pituitary gland. When bone marrow aspiration was performed, lymphoma infiltration to the bone marrow was detected and the thrombocytopenia was thought to be resulting from lymphoma infiltration of the bone marrow. Chemotherapy and radiotherapy to the sellar area were planned, but the patient died and her family did not permit an autopsy.

## Discussion

We have described a patient with non-Hodgkin's lymphoma infiltration of the pituitary gland, who presented with ophthalmoplegia and DI.

Sudden onset of DI, ophthalmoplegia and headaches in a patient over 50 years of age should always raise suspicion for lymphoma or leukemia of the pituitary gland or metastasis to the pituitary. The vast majority of central nervous system (CNS) lymphomas are non-Hodgkin's B-cell lymphomas as in our patient, and CNS lymphomas are usually observed in patients with acquired immunodeficiency syndromes [2].

As the pituitary gland has a great reserve capacity, pituitary metastasis and pituitary involvement in lymphoma or leukemia appear to be asymptomatic in most cases [4]. Metastasis to the pituitary and pituitary involvement in lymphoma or leukemia manifests itself as DI. Diabetes insipidus seems to be the most common clinical presentation; this correlates with the observation that metastatic and infiltrative diseases of the pituitary preferentially involve the posterior lobe. The predilection of tumor involvement of the posterior pituitary may be because the posterior lobe of the pituitary is supplied with blood directly from the systemic circulation while the anterior lobe is not [4]. The posterior lobe is supplied by the hypophyseal arteries [4] whereas the anterior lobe is nourished by the portal vessel system and secondarily by the lower infundibular stem which partly arises from the posterior lobe [1,4,5]. Anterior pituitary involvement can also occur as a result of extension of the tumor from the posterior pituitary, the hypothalamus or bony walls of the sella. There are, however, reports of isolated tumor deposits in the anterior lobe of the pituitary without evidence of involvement of the surrounding structures [3,4]. Involvement of the pituitary associated with lymphoma is very uncommon compared to that of secondary spread from solid tumors [5-7]. In our patient, primary lymphoma was in the sphenoid sinus, and pituitary involvement occurred as a result of extension of the lymphoma infiltration of the bony walls of the sella.

Occasionally DI is transient, because regeneration of neuro-hypophyseal fibers may occur or intermittent corticotrophin cell insufficiency may obliterate the presence of DI until corticosteroid treatment is instituted. Hypothyroidism and hypoadrenalism are the most frequent types of symptomatic hypopituitarism. Bilateral hemianopsia is the most common type of visual field impairment. Infiltration of the adjacent cavernous sinus usually induces cranial nerve III palsy or less frequently nerve IV palsy; compression of nerve VI is relatively uncommon because it is well sheltered within the cavernous sinus. Facial numbness due to cranial nerve V dysfunction is also rare. Tumor extension to the septum pellucidum or the frontal lobes may result in cognitive deficit or psychiatric symptoms and anosmia. If cranial nerve I is affected, stretching of the diaphragm sella or ventricular distention can give rise to headaches or intracranial hypertension [5]. In our patient, cranial nerves III, IV and VI were induced.

Hyperprolactinemia is attributed to stalk compression; the degree of hyperprolactinemia is important for the differential diagnosis, because PRL levels above 200 ng/ml (9.0 nmol/liter) are generally considered to be indicative of a prolactinoma. The prolactin level of our case was consistent with the prolactin levels observed in the presence of stalk compression [5,7].

The differential diagnosis must be established with pituitary adenoma and meningioma which are the most frequent tumors of the pituitary gland. Although symptoms of lymphoma or leukemia infiltration of the pituitary gland and metastasis to the pituitary at presentation may resemble pituitary adenomas, DI is reported in only 1% of adenomas [5,8].

Schubiger and Haller suggested that DI is the most important criterion to differentiate pituitary adenomas from tumor metastasis and infiltration of lymphoma or leukemia to the pituitary [5].

Presentation with hypopituitarism, headaches or visual disturbances is less helpful in the differential diagnosis, being described in infiltration of the pituitary gland with lymphoma, leukemia and metastasis to the pituitary. Ophthalmoplegia may be more helpful in the differential diagnosis, without being pathognomonic for metastasis either [5,9].

In contrast, presentation with abducens nerve palsy should always raise the suspicion for malignancy. The rapidity of symptom development may also contribute to the diagnosis. Consequently, a rapidly growing sella tumor, especially in patients treated with dopamine agonists or a sudden onset of DI, ophthalmoplegia and headaches in a patient of more than 50 years of age as reported here, strongly suggest lymphoma or leukemia, infiltration of pituitary or metastasis to the pituitary gland [5].

Differential diagnosis must be established for the frequent tumors of the sellar area such as pituitary adenoma and meningioma. High resolution CT and MRI are more sensitive for the differential diagnosis. Typical MR findings of CNS lymphoma are mass lesions that are iso-hypointense on T_1 _and T_2 _weighted images. The absence of T_2 _prolongation results from the dense cellularity and high nucleus to cytoplasm ratio of lymphoma, which may help in the differentiation of CNS lymphoma from other brain tumors [2,10,11].

Typically, contrast enhancement is intense and homogeneous in immunocompetent patients, but it is more likely to be non-homogeneous or ring-like in immunocompromised patients [2]. Pituitary adenoma and meningioma may exhibit MR signal intensity characteristics that are similar to those of lymphoma, but they do not spread perineurally, and do not usually have a long dural tail. Metastasis to the pituitary gland was the second consideration of our differential diagnosis on MR images. Metastasis usually appears as a dumbbell-shaped intrasellar and suprasellar tumor with only a small indentation at the level of the diaphragm sella or as a suprasellar tumor that invades rather than displaces the infundibular recess of the third ventricle; metastasis usually does not exhibit sellar enlargement because of the relatively rapid tumor growth. The diagnosis is made definite by biopsy and histopathological examination [1,2]. Non-Hodgkin's lymphoma in elderly patients carries a notoriously poor prognosis. Our patient died before chemotherapy and radiation therapy could be started; however, even with chemotherapy and radiation therapy, the chance of remission is very low. We could not learn the exact reason for the death as the family of the patient did not permit an autopsy. The choice of treatment for localized cerebral non-Hodgkin's lymphoma is radiation therapy; however, cerebral radiation carries a risk of deteriorating cortical function, brain atrophy and leukoencephalopathy, especially in older patients [3,12].

## Conclusion

Lymphoma infiltration to the pituitary is difficult to differentiate from pituitary adenoma, meningioma and other sellar lesions. But when iso-hypointense mass lesions are present on T_1 _and T_2 _weighted images of an older patient with immunodeficiency, lymphoma infiltration of the pituitary must be considered. To plan the treatment of lymphoma infiltration of the pituitary gland, it must be differentiated from other sellar lesions.

## Abbreviations

ACTH: adrenocorticotrophic hormone; CNS: central nervous system; DI: diabetes insipidus; MRI: magnetic resonance imaging; CT: computed tomography; FSH: follicle-stimulating hormone; LH: luteinizing hormone; PRL: prolactin; TSH: thyroid stimulating hormone; BUN: blood urea nitrogen; WBC: white blood cell count.

## Competing interests

The authors declare that they have no competing interests.

## Authors' contributions

GT was the attending physician of the case and the diagnosis was established by her under the supervision of FA. This case report was written by GT and partly by IK and overviewed by FA.

## Consent

Written informed consent was obtained from the patient's next of kin for publication of this case report and any accompanying images. A copy of the written consent is available for review by the Editor-in-Chief of this journal.
